# Novel High-Throughput DNA Part Characterization Technique for Synthetic Biology

**DOI:** 10.4014/jmb.2207.07013

**Published:** 2022-07-26

**Authors:** Seong-Kun Bak, Wonjae Seong, Eugene Rha, Hyewon Lee, Seong Keun Kim, Kil Koang Kwon, Haseong Kim, Seung-Goo Lee

**Affiliations:** 1 Synthetic Biology Research Center, Korea Research Institute of Bioscience and Biotechnology, Daejeon 34141, Republic of Korea; 2 Biosystems and Bioengineering Program, University of Science and Technology, Daejeon 34141, Republic of Korea

**Keywords:** Synthetic biology, DNA parts, circuit design, long-read sequencing, image analysis

## Abstract

This study presents a novel DNA part characterization technique that increases throughput by combinatorial DNA part assembly, solid plate-based quantitative fluorescence assay for phenotyping, and barcode tagging-based long-read sequencing for genotyping. We confirmed that the fluorescence intensities of colonies on plates were comparable to fluorescence at the single-cell level from a high-end, flow-cytometry device and developed a high-throughput image analysis pipeline. The barcode tagging-based long-read sequencing technique enabled rapid identification of all DNA parts and their combinations with a single sequencing experiment. Using our techniques, forty-four DNA parts (21 promoters and 23 RBSs) were successfully characterized in 72 h without any automated equipment. We anticipate that this high-throughput and easy-to-use part characterization technique will contribute to increasing part diversity and be useful for building genetic circuits and metabolic pathways in synthetic biology.

## Introduction

In synthetic biology, the term DNA part refers to a DNA fragment with a specific function. Each fragment is composed of a promoter, ribosome-binding site (RBS), terminator, and insulator that together regulate the expression of the coding DNA sequence. Such characterized DNA parts and the standardized assembly methods provide the basis of optimized genetic circuits or metabolic pathways. DNA part characterization is commonly defined as quantification of expression rate of the part. Determining the quantitative characteristics of DNA from a given strain is important for designing and constructing a predictable genetic circuit. To this end, synthetic biologists have characterized the biologically standardized DNA parts and stored the information in databases such as the iGEM Parts Registry [1-4]. Currently, the Registry holds data on more than 20,000 identified DNA parts, but many are yet to be characterized, and those that have been characterized are mostly limited to *E. coli*. Thus, a rapid characterization method is required to quantify the expression of the DNA parts that could be available from many different host strains [5-7].

In general, it is difficult to measure the attributes of hundreds of thousands of DNA parts as well as determine optimal culture conditions for the parts. The most well-known DNA part characterization method is the measurement of the relative strength of the DNA part through the expression of fluorescent proteins. This method involves manipulating the genotype (including the given DNA part) for fluorescent protein expression and subsequent cloning and characterization of the part’s strength based on its corresponding phenotype (measured via fluorescence intensity). However, this method requires expensive devices such as flow cytometers to measure single-cell level signals. In addition, the time taken for cloning and single-cell fluorescence measurements increases exponentially as the number of DNA parts increases. Moreover, if the respective DNA parts are used in a different host strain, the strength of the parts may change from what was determined originally, demonstrating the need to develop a technique to rapidly characterize multiple DNA parts in many different strains [8, 9]. In this study, we developed an imaging-based, high-throughput DNA part characterization technique that uses solid plate-based phenotyping and in-house, long-read sequencing techniques [10-12].

## Materials and Methods

### Strain and DNA Preparation

*Escherichia coli* DH5α, BL21, and C2566 were purchased from New England Biolabs (USA). *E. coli* DH5α was used for DNA preparation and cloning. The cells were grown in lysogeny broth (LB) (Difco Laboratories Inc.,USA) with appropriate antibiotic supplementation (25 ug/ml kanamycin and 34 ug/ml chloramphenicol) for strain maintenance and plasmid construction in the different *E. coli* strains. Three *E. coli* strains (DH5α, BL21, C2566) were used for part characterization, which was conducted at 37°C on LB agar plates with optimum time-course for colony formation. The sequences of the DNA parts used in this study are presented in Table S1. The promoter and RBS were selected from the Registry (http://parts.igem.org) and the terminators were from [4]. For the DNA part preparation, the duplex oligosynthesis method of Macrogen (Korea) was employed.

### Golden Gate Assembly for Combinatorial Library Assembly

For Golden Gate assembly, the selected DNA parts were designed with the addition of a BsaI-specific site (GGTCTC) on either end of the sequence as well as a 1 bp spacer sequence (**A**) for enzyme function and a 4 bp designed overhang sequence for each part type (Table S2). Golden Gate assembly was performed using a modified pACBB vector that had an inserted BsaI site [13, 14]. Five stretches of 4 bp sticky end overhangs were selected considering the DNA ligation efficiency to allow up to four different DNA parts to assemble in the destination vector (pACBB) with high accuracy [15]. For the Golden Gate assembly reaction, 56 fmol of DNA part solution was added to 112 fmol of destination vector solution, and the volume of the DNA solution amounted to 16 μl using DW. Next, 1 μl of BsaI_HFv2 (NEB #R3733), 1 μl of T4 DNA ligase (HC), and 2 μl of T4 DNA ligase (HC) buffer (Promega, USA) were added to the DNA to create a 20 μl reaction solution. The DNA concentrations were measured using a Qubit Fluorometer 4 (Invitrogen, USA) and a 1× dsDNA HS Assay Kit (Invitrogen).

### High-Throughput Phenotyping with Fluorescent Colony Image

The combinatorial part characterization circuit library assembled via Golden Gate cloning was transformed into each *E. coli* strain and then grown in agar plates. Images of the entire culture plate were taken with a Nikon fluorescence microscope (Nikon Instruments Inc., Japan). The green fluorescent protein (GFP) signals were photographed with the green light filter (excitation: 470 nm, emission: 520 nm). The red fluorescent protein (RFP) signals were photographed with the red light filter (excitation: 540 nm, emission: 605 nm). Both fluorescent signals were photographed at optimum exposure time determined with consideration to the dynamic range. The fluorescent images were analyzed for the position, size, and RGB value of each colony using “OpenCFU” [16]. The position value was used to obtain the index through which a Python script was used to connect the colonies using the RFP and GFP images. Next, for each indexed colony, the red channel value and size were calculated as the total RFP, and the green channel value and size were calculated as the total GFP for characterization.

### Assembled Plasmid Library Sequencing

The circular DNA library prepared through Golden Gate assembly was immediately treated using the Rapid Barcoding Sequencing protocol from Oxford Nanopore Technologies (ONT, UK). The DNA concentrations were determined using a Qubit fluorometer and then increased to suitable levels using an AMpure XP Bead (Beckman Coulter, USA). The complete sequencing library was loaded onto an R9.4.1 Flongle Flow Cell (ONT) for sequencing in a MiniON MK1C device. The raw sequencing data were converted and pre-processed into fastq files with NanoFilt, the guppy basecaller (v5.0.1) from ONT [17, 18]. To verify the DNA parts of each selected read, minimap2 was used for multi-reference mapping of the reference sequence, which included all DNA part sequences available for combination [19]. For the final result, primary mapping data were selected using SAMtools [20]. The “pysam” package of Python software (v3.7.0) was used to determine the reads at ≥ 95% query coverage. With upper processed reads, the count number of reads by each genotype and profile for the whole sequencing library was based on the ratio for each genotype.

### Pooled Colony Genotyping with Tagged Sequencing

The GFP module of the characterization circuit was amplified for sequencing through colony PCR using AccuPower PCR Premix (Bioneer, Korea). The colony PCR was performed using the forward and reverse tag primers that bound the tagging primer-binding site at either end of the GFP module. The tag primer consists of a 7 bp sequence as a tagged barcode and a 17–20 bp binding sequence. To produce the tag sequence, the “create.dnabarcodes” of the “DNABarcodes” package in R Bioconductor was used [21]. Eight forward and twelve reverse primers were designed to confer different tag sequence combinations to as many as 96 colonies. The tag primer sequences are presented in Table S3. The tag-attached amplified DNA generated via colony PCR was collected in a single tube for sequencing. The pooled amplified DNA samples were prepared using the Ligation Sequencing Kit (ONT). Running sequencing and pre-processing methods were the same as described above. Selected reads were demultiplexed by their own forward and reverse tag using R software (v4.0.5) with “VmatchPattern” of the R Bioconductor “Biostrings” package [22].

### Calculating the Relative DNA Part Unit

The quantified values of the DNA parts were expressed as relative promoter unit (RPU) and relative RBS unit (RRU), respectively, in reference to [5]. For the calculation, the promoter (J23119) and RBS (B0030) were used as the standard promoter and RBS, respectively. These standard parts were cloned and the standard characterization circuit (J23119 – B0030 – sfGFP – L3S2P56) was prepared. The colony fluorescence intensity of the standard circuit was used as the baseline. The promoter or RBS of the standard circuit was replaced with the part to be measured, and the colony fluorescence intensity was measured to calculate the relative promoter unit or relative RBS unit (RPU and RRU, respectively) as follows:

$$\text{RPU or RRU}=\frac{\text{Average colony fluorescence unit}}{\text{Standard circuit's colony fluorescence unit-Average colony fluorescence unit}}$$ (3)

## Results

### Building a DNA Part Characterization Library

**Fig. 1:**
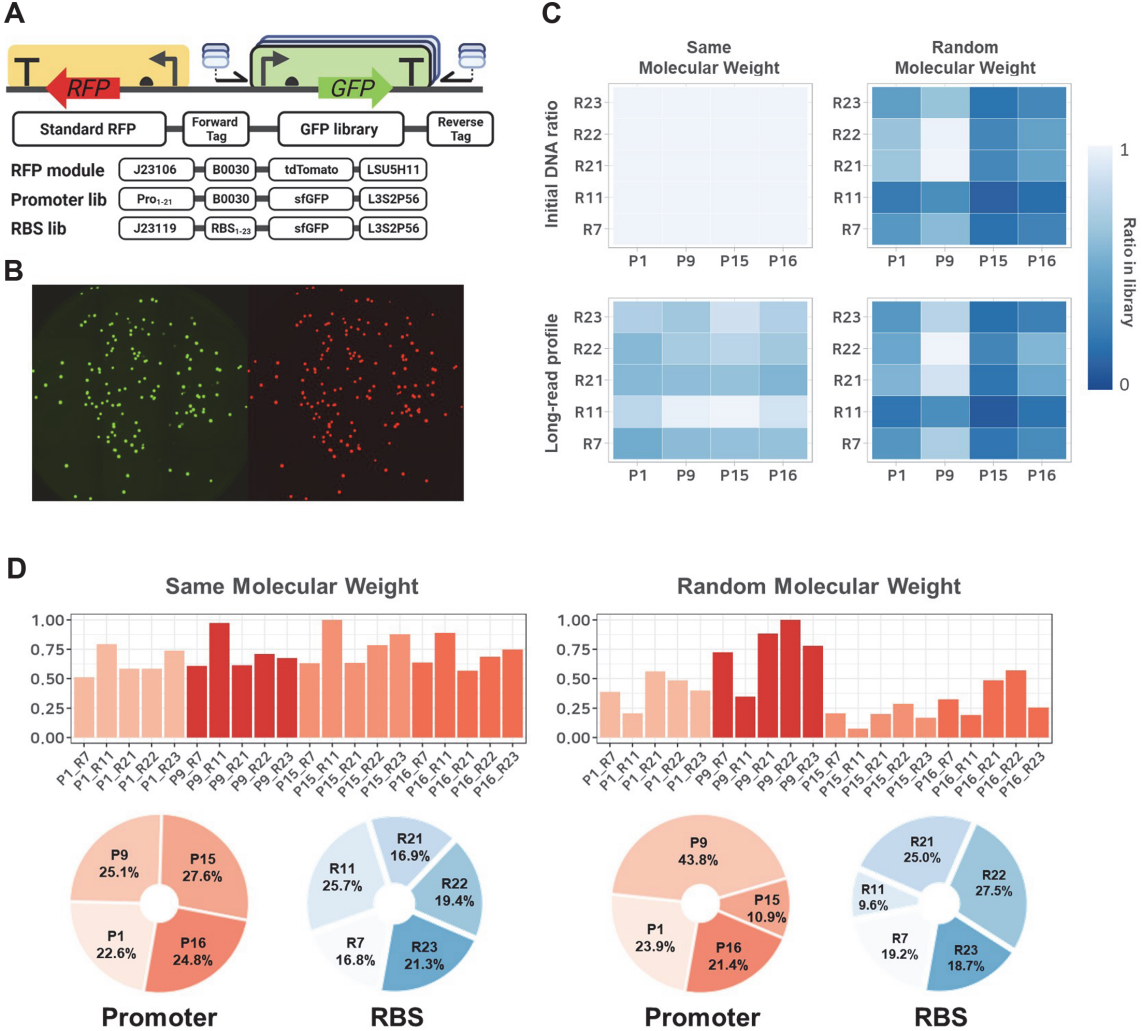
Combinatorial part library profiles using long-read sequencing. (**A**) Simple diagram of combinatorial DNA library for part characterization. The part details used for each standard circuit are described below the circuit diagram. (**B**) Fluorescent image of cells containing standard characterization circuit which expresses GFP and RFP. (**C**) Heatmap of the input DNA part molar ratio in the two combinatorial libraries and measured DNA part profiles from long-read sequencing. The promoters in combination are presented horizontally, and RBS is presented vertically. (**D**) Details of the DNA part profiles by analyzing long-read sequencing. The barplots show the part combination ratio of the two combinatorial libraries, with the yaxis representing its ratio in the library and the x-axis representing the promoter-RBS combinations. The pie charts at the bottom show the ratio of the part (promoter, RBS) in the libraries.

A genetic circuit for part characterization was constructed to quantify part strength by fluorescent signal measurements. The part characterization circuit consisted of GFP and RFP modules for fluorescence-based phenotyping and a tag primer-binding site for genotyping (Fig. 1A). The GFP module responsible for the quantifiable fluorescence signals was generated by a combinatorial part library. The RFP module was expressed as a single fixed combination to account for the effects of microbial growth rate [5]. The two modules were designed in opposite directions to minimize the transcriptional read-through effect. Two fluorescent proteins, sfGFP and tdTomato, were selected as they showed minimum interference and similar ranges of fluorescence intensity [23, 24]. The tag primer-binding site was used to attach a tag sequence which allows pooling of differently tagged colonies into one sample for high-throughput sequencing. The combinatorial part library was constructed using Golden Gate assembly and inserted into the GFP module while the RFP module contained a single fixed combination of the part (Fig. 1B). To investigate the part library, two types of part libraries were prepared with four promoters, five RBSs, and one terminator. The names of the parts are presented as numbers in Table S1. One library used the same molar ratio for all DNA parts of each type. The other used random molar ratios for all parts of each type. We used a nanopore long-read sequencing technique and profiled the distribution of the assembled DNA parts [25, 26]. The results showed that the frequency of DNA part combination was strongly positively correlated with the molar ratio of each part introduced in the assembly (Figs. 1C and 1D). Also shown is that evenly distributed combinatorial part libraries can be built when the parts were combined at an identical ratio.

### High-Throughput Phenotyping on a Solid Plate

**Fig. 2:**
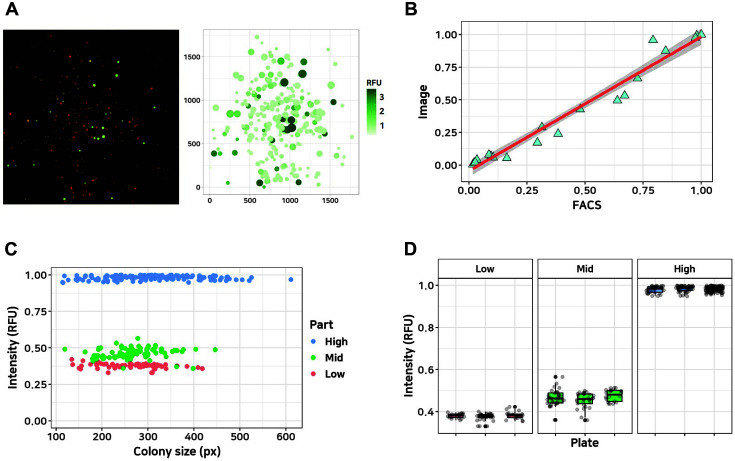
Image-based high-throughput phenotyping. (**A**) Indexed colony image (left) and converted fluorescent data by computational method (right). The shade green (RFU) of the dot shows colony intensity, and the size of the dot represents colony size. (**B**) Comparison of fluorescent values using single cell-based FACS and the microscopy-based method developed in this study. *x*- and *y*-axes represent the values from FACS and microscopy, respectively. The red line is the regression-fitted line with a 95% confidence interval (gray). (**C**) Examination of colony size-driven bias. Blue, green, and red represent high-, middle-, and low-intensity colonies in the same plate, respectively. The *x*-axis represents colony size (number of pixels in image), and the *y*-axis represents colony intensity calculated with Eq. (1). (**D**) Biological replicates with low-, middle-, and high-intensity parts performed on different plates on different days. Each boxplot shows the mean and deviation calculated with colonies on different plates.

For rapid phenotyping of the combinatorial part library, we used an imaging-based computerized method that can obtain the fluorescence value of each colony on a solid plate. The combinatorial library was transformed into *E. coli* strains, which were grown on agar plates. The fluorescent images of each colony were analyzed for the position, size, and RGB value using “OpenCFU.” The fluorescence intensity of each colony (colony intensity) containing the characterization circuit was defined as the intensity of GFP based on RFP (Eq. 1). Fig. 2A shows the colony image and the calculated colony intensity with a scatter plot.

$$\text{Colony intensity}=\frac{\text{Green channel value}\times \text{Colony size in green channel}}{\text{Red channel value}\times \text{Colony size in red channel}}$$ (1)

To evaluate the proposed method, twenty colonies harboring the part characterization circuit were analyzed using a fluorescent microscope as well as a conventional flow cytometry single cell analytic device. Fig. 2B shows that the two groups of measured values were strongly positively correlated. To examine cell variation and reproducibility of the image analysis, three characterized circuits with varying GFP expression were transformed into *E. coli* and more than 100 colonies from each sample were investigated using their GFP fluorescence. Figs. 2C and 2D show that the standard deviation was low between the minimum (5.2%) and maximum (7.7%), while the colony size on the plate was found to not influence the quantified values.

### Multiplexed Genotyping with Nanopore Long-Read Sequencing

**Fig. 3:**
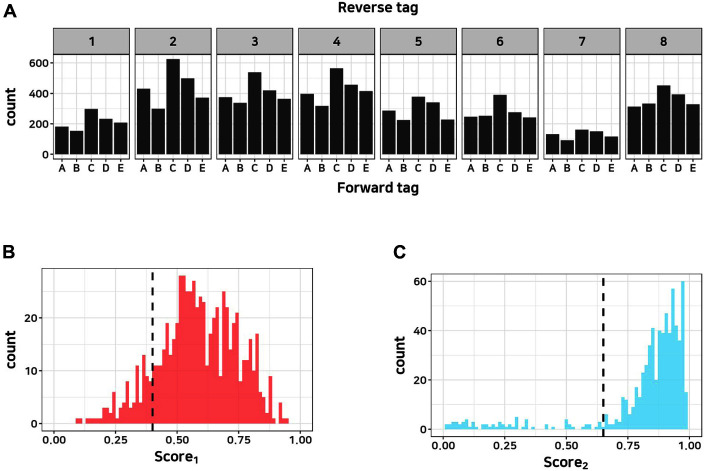
Evaluation of multiplexed genotyping. (**A**) Read count distribution for each barcoding sample. Each number on each top of the graph and alphabetical value represents a reverse and forward tag, respectively. The *y*-axis represents the number of reads demultiplexed with the specific tag combination. (**B**) The histogram of tag score1 values. The *y*-axis represents the number of tags. (**C**) The histogram of tag score2 values. It shows that most of the tagged data satisfy the empirical QC criteria.

Solid plate-based phenotyping can be conducted in a high-throughput manner, but subsequent genotyping of the colonies is a time-consuming and labor-intensive process, which inevitably slows down the workflow of part characterization. To increase the throughput of genotyping of multiple colonies, designed barcode tags were attached to each combinatorial DNA sequence in the GFP modules by using a colony PCR technique. The barcode tags enable sequencing of all the DNA sequences at once by pooling the colony PCR products. After phenotyping through imaging, the GFP module sequence was amplified through tagged colony PCR with 8 forward and 12 reverse primers to generate up to 96 barcode sequence combinations. This multiplexing number can increase up to 2304 (ONT barcodes 횞 forward tags 횞 reverse tags), as the additional 24 barcodes provided by ONT could be used. In this study, we picked around 200 colonies manually and their amplified DNAs were pooled in a single tube after tag attachment with 5 forward and 8 reverse tags. The complete sequencing library was loaded to an R9.4.1 Flongle Flow Cell for sequencing in a MiniON MK1C device. Fig. 3A shows the numbers of reads for the barcode-tagged samples after demultiplexing the pooled sequencing results. Despite some bias, each sample had more than 200 reads, which is enough to further analysis. To evaluate the demultiplexing of the reads, the quality score_1_ and score_2_ were calculated using the “qcat” program, an official demultiplexing program of ONT (Eq. 2). Figs. 3B and 3C show the tagged data satisfying the empirical QC criteria (Total read count > 15, score_1_ > 0.4, score_2_ > 0.65).

$$\begin{array}{l} Score_{1}=\frac{\mathrm{Pr}imary\;mapped\;reference\;read\;count}{Total\;read\;count}\\ Score_{2}=\frac{\mathrm{Pr}imary\;mapped\;reference\;read\;count\;-\;\mathrm{Sec}ond\;mapped\;read\;count}{\mathrm{Pr}imary\;mapped\;reference\;read\;count} \end{array}$$ (2)

### Promoter and RBS Characterization in Three *E. coli* Strains

**Fig. 4:**
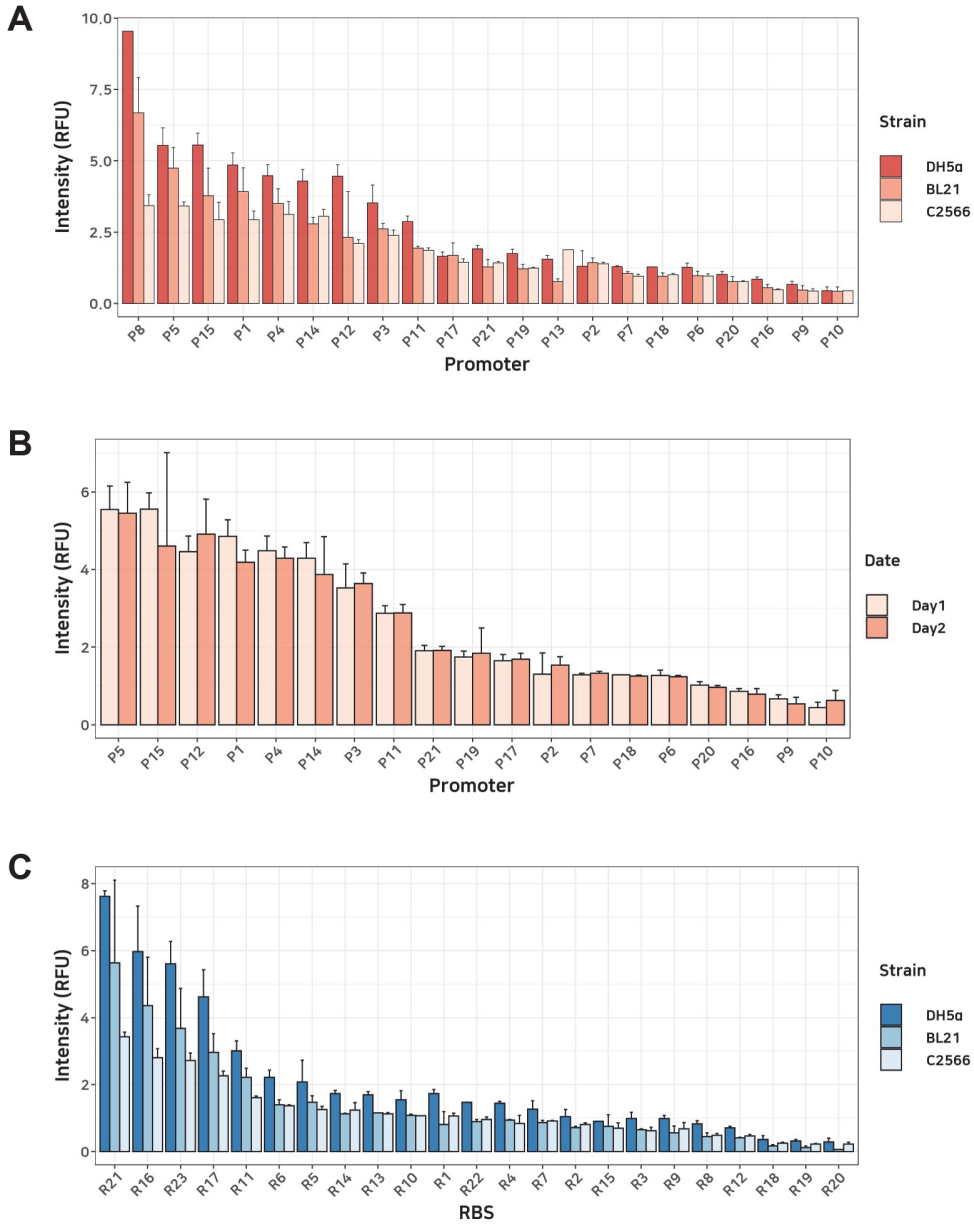
Part strengths of the promoter and RBS libraries. (**A**) RFU of the promoter library on three different *E. coli* strains. (**B**) Comparisons between the biological replicates of the promoter library. The biological replicates of 2 promoters could not be obtained, so they were removed. (**C**) RFU of the RBS library on three different *E. coli* strains. All results were obtained from 240 colonies according to strain and part type (promoter or RBS). Colonies were obtained from three different 9 cm plates generated at the same time.

In this study, we characterized a total of 44 *E. coli* DNA parts (21 promoters and 23 RBSs). Each type of DNA part was assembled into the part characterization circuit. Each of the 21 promoters and 23 RBSs replaced the standard promoter and RBS region of the part characterization circuit, respectively, through Golden Gate assembly. The library was transformed into three different strains of *E. coli* (DH5α, BL21, and C2566) in order to compare strain-specific part characteristics. Considering the different growth rates for each strain, the colonies were imaged immediately after formation. We picked more than 200 colonies randomly and sequenced them for genotyping. Colony intensity can be determined for between three and twenty colonies for each part, and the mean of the colony intensity was used to calculate the part intensity (RFU). Figs. 4A and 4B show that the intensities of each part are comparable among the three strains, which indicates that strong promoters in a specific strain also show strong characteristics in the other strains. The intensities tend to be higher in the *E. coli* K DH5α strain compared to the B strains (BL21 and C2566). We confirmed that the entire procedure of these 44 part-characterizations with three strains could be done by a graduate student-level researcher within 72 h excluding the part preparation steps.

## Discussion

This study introduces a high-throughput DNA part characterization method based on imaging and long-read sequencing techniques. Our method is advantageous as it allows the characterization of multiple DNA parts rapidly with commonly used imaging tools such as a low-cost fluorescent microscope instead of high-cost devices, such as a microplate reader or flow cytometer used in conventional methods. ONT sequencing devices are also increasingly available in small-scale laboratories at much lower cost than conventional NGS devices. We were able to characterize 21 promoters and 23 RBSs in approximately 72 h in three *E. coli* strains (DH5α, BL21, and C2566), a task that could take more than two weeks to be completed by cloning multiple parts individually (Table S1).

It is necessary to compute the absolute unit and the relative unit of a DNA part in the context of a large set of possible DNA part combinations since the strength of a DNA part can change significantly as its combinatorial DNA partners are changed. The combination of such parts in set conditions may increase exponentially as the parts increase in number, while the use of a few well-defined parts could increase the functional instability due to the increase in the proportion of repeated sequences within the system [27]. Our approach using combinatorial library assembly, high-throughput phenotyping, and pooled genotyping is able to speed up part characterization and promote efficiency in synthetic biology research, especially in the field of genetic circuit design and prediction [28-31].

**Fig. 5:**
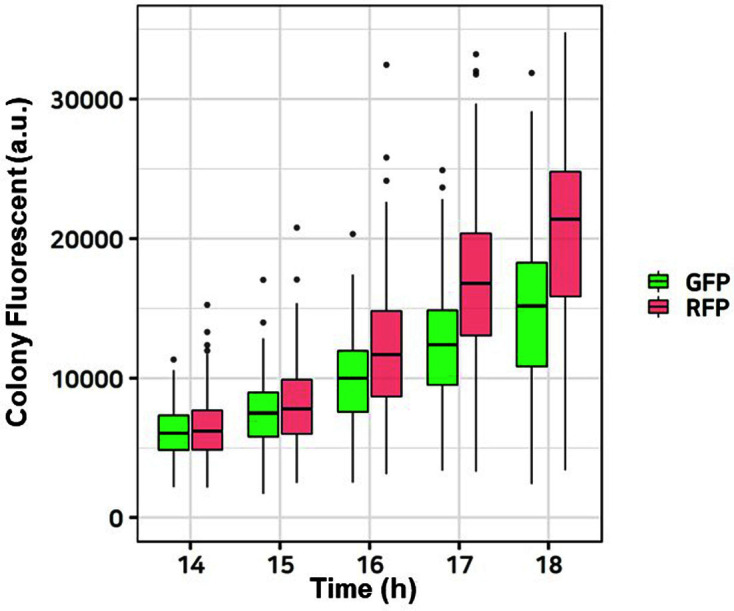
Maturation time of fluorescent proteins. Time-course fluorescent values of GFP and RFP used in this study were measured on plates. The *y*-axis and *x*-axis represent the fluorescent value of the colonies and time (h), respectively; extra black dots represent outliers.

However, a more accurate strategy is required for measuring fluorescence in different strains due to their differences in colony formation, fluorescent protein expression, maturation times, and growth rate (Fig. 5). In addition, pixel saturation (where the measurement capabilities of the imaging device were exceeded due to too strong or too weak fluorescence values) was an issue [32]. This issue occurs when various colonies with large deviations in fluorescence coexist on a single plate, which is common for libraries with varying genotypes. The light intensity that allows photographing in imaging analysis is influenced by the exposure time, which also influences the range of measurable fluorescence. If the exposure time is too short, the fluorescence of weak intensity cannot be measured, and if the exposure time is too long, the actual fluorescence exceeds the level of measurable fluorescence, causing pixel saturation and resulting distortion. In this study, an exposure time of 0.3 s was selected as our empirical criterion. The novel techniques developed in this study are anticipated to contribute to expanding the capabilities of DNA part characterization beyond *E. coli* in synthetic biology. The quantified promoters and RBSs can be generally used to build a library of a metabolic pathway for its optimal metabolic reactions. We can also use the well- characterized parts to design a logic gate circuit that can control multiple genes, for example, the expressions of more than eight genes can be controlled by using just three input inducers. The imaging-based fluorescence measurements and DNA library profiling through long-read sequencing for colony sequencing are also expected to be highly useful for other synthetic biology endeavors such as designing biosensors with fluorescent protein reporters and DNA enzyme activity measurements. As further advancements are made in synthetic biology, characterized parts shared among researchers in many fields of synthetic biology will contribute to the synthetic biology ecosystem.

## Supplemental Materials

Supplementary data for this paper are available on-line only at http://jmb.or.kr.
