# Supplementary material for: Novel High-Throughput DNA Part Characterization Technique for Synthetic Biology
Source: J Microbiol Biotechnol. 2022 Jul 26;32(8):1026–33. doi: 10.4014/jmb.2207.07013 (PMC9628936; doi:10.4014/jmb.2207.07013)
Supplement: Supplementary file 1 [file jmb-32-8-1026-supple.pdf]

J. Microbiol. Biotechnol.
https://doi.org/10.4014/jmb.2207.07013

Supplementary Tables

Supplementary Table 1. List of DNA parts in this study

Name	Sequence (5'→3')	Intensity (RFU)	ID	Type
BBa_I14018	TGTAAGTTTATACATAGGCGAGTACTCTGTTATGG	4.520	P1	Promoter
BBa_I14034	CATTATTGCAATTAATAAACAACTAACGGACAATTCTACCTAACA	1.471	P2	Promoter
BBa_J23101	TTTACAGCTAGCTCAGTCCTAGGTATTATGCTAGC	3.553	P3	Promoter
BBa_J23104	TTGACAGCTAGCTCAGTCCTAGGTATTGTGCTAGC	4.399	P4	Promoter
BBa_J23111	TTGACGGCTAGCTCAGTCCTAGGTATAGTGCTAGC	5.516	P5	Promoter
BBa_J23115	TTTATAGCTAGCTCAGCCCTTGGTACAATGCTAGC	1.265	P6	Promoter
BBa_J23116	TTGACAGCTAGCTCAGTCCTAGGGACTATGCTAGC	1.307	P7	Promoter
BBa_J23119	TTGACAGCTAGCTCAGTCCTAGGTATAATGCTAGC	9.542	P8	Promoter
BBa_K088007	TATAAGATCATACGCCGTTATACGTTGTTTACGCTTTG	0.601	P9	Promoter
BBa_K119000	TTTACACTTTATGCTTCCGGCTCGTATGTTGTGTGGAC	0.548	P10	Promoter
BBa_M13101	CCCGTCTAATGCGCTTCCCTGTTTTTATGTTATTCTCTCTGTAAAGG	2.873	P11	Promoter
BBa_M13104	TTGATAAATTCACTATTGACTCTTCTCAGCGTCTTAATCTAAGCTATCG	4.684	P12	Promoter
BBa_M13106	GGTAAACCATATGAATTTTCTATTGATTGTGACAAAATAAACTTATTCC	1.551	P13	Promoter
BBa_M13108	AATCTCCGTTGTACTTTGTTTCGCGCTTGGTATAATCGCTGGGGGTC	4.066	P14	Promoter
BBa_M13110	TCTTTTTGATGCAATCCGCTTTGCTTCTGACTATAATAGTCAGGGTAA	5.172	P15	Promoter
BBa_R1075	TTAAATTTCCTCTTTTCAGGCCGGAATAACTCCCTATAATGCGCCACCA	0.829	P16	Promoter
BBa_J23100	TTGACGGCTAGCTCAGTCCTAGGTACAGTGCTAGC	1.672	P17	Promoter
BBa_J23106	TTTACGGCTAGCTCAGTCCTAGGTATAGTGCTAGC	1.263	P18	Promoter
BBa_J23109	TTTACAGCTAGCTCAGTCCTAGGGACTGTGCTAGC	1.801	P19	Promoter
BBa_J23114	TTTATGGCTAGCTCAGTCCTAGGTACAATGCTAGC	1.002	P20	Promoter
BBa_J23117	TTGACAGCTAGCTCAGTCCTAGGGATTGTGCTAGC	1.913	P21	Promoter
BBa_J61100	TCTAGAGAAAGAGGGGACAAA	1.736	R1	RBS
BBa_J61101	TCTAGAGAAAGACAGGACCCA	1.041	R2	RBS
BBa_J61104	TCTAGAGAAAGAAGGGACAGA	0.984	R3	RBS
BBa_J61106	TCTAGAGAAAGATAGGAGACA	1.447	R4	RBS
BBa_J61110	TCTAGAGAAAGAGGCGAATTA	2.076	R5	RBS
BBa_J61113	TCTAGAGAAAGAGTGGAAAAA	2.218	R6	RBS

BBa_J61116	TCTAGAGAAAGACATGAGGCA	1.266	R7	RBS
BBa_J61121	TCTAGAGAAAGAGACGAGTCA	0.829	R8	RBS
BBa_J61125	TCTAGAGAAAGAGCCGAGTTA	0.986	R9	RBS
BBa_J61127	TCTAGAGAAAGAGTGGAACTA	1.547	R10	RBS
BBa_J61132	TCTAGAGAAAGACAGGATTAA	3.009	R11	RBS
BBa_J61135	TCTAGAGAAAGACCGGAGACA	0.703	R12	RBS
BBa_J61137	TCTAGAGAAAGAGTAGATCAA	1.690	R13	RBS
BBa_B0029	TCTAGAGTTCACACAGGAAACCTA	1.733	R14	RBS
BBa_B0033	TCTAGAGTCACACAGGACTA	0.905	R15	RBS
BBa_B0035	TCTAGAGATTAAAGAGGAGAATA	5.972	R16	RBS
BBa_B0064	TCTAGAGAAAGAGGGGAAATA	4.620	R17	RBS
BBa_B0072	TCTAGAGCACCACTA	0.363	R18	RBS
BBa_B0073	TCTAGAGTCACACCACTA	0.320	R19	RBS
BBa_B0074	TCTAGAGTCACACCACCCTA	0.288	R20	RBS
BBa_B0030	TCTAGAGATTAAAGAGGAGAAATA	7.617	R21	RBS
BBa_B0032	TCTAGAGTCACACAGGAAAGTA	1.475	R22	RBS
BBa_B0034	TCTAGAGAAAGAGGAGAAATA	5.608	R23	RBS
L2U5H11	TAGCGTGCGAACAGCACGCTATTGTTGTAT		T1	Terminator
L3S1P56	TTTTCGAAAAAAGGCCTCCCAAATCGGGG GGCCTTTTTTATTGATAACAAAA		T2	Terminator

* RFU is calculated in *E.coli* DH5α

Supplementary Table 2. Overhangs for golden gate assembly

Sequence (5'→3')	Description	Reference
GCCT	Vector – Promoter	Potapov et al. 2018
CTTT	Promoter – RBS	Potapov et al. 2018
GCAG	RBS – CDS	Potapov et al. 2018
CTAA	CDS – Terminator	Potapov et al. 2018
TCAC	Terminator - Vector	Potapov et al. 2018

Supplementary Table 3. Tag primer sequence for tagging colony PCR

Name	Sequence (5'→3')	Type
p15A_ori_A	GCATGTACAGAGCAAGAGATTACGCGC	Forward
p15A_ori_B	TTGTCTCCAGAGCAAGAGATTACGCGC	Forward
p15A_ori_C	GTTCGAACAGAGCAAGAGATTACGCGC	Forward
p15A_ori_D	TGTCGAGCAGAGCAAGAGATTACGCGC	Forward
p15A_ori_E	TAGGCTTCAGAGCAAGAGATTACGCGC	Forward
p15A_ori_F	ACATAGGCAGAGCAAGAGATTACGCGC	Forward
p15A_ori_G	GACTTACCAGAGCAAGAGATTACGCGC	Forward
p15A_ori_H	ATGGTCCCAGAGCAAGAGATTACGCGC	Forward
M13F_1	TGGACTAGTAAAACGACGGCCAGT	Reverse
M13F_2	ATCAACCGTAAAACGACGGCCAGT	Reverse
M13F_3	ACCAGAAGTAAAACGACGGCCAGT	Reverse
M13F_4	GATCTCCGTAAAACGACGGCCAGT	Reverse
M13F_5	CTAAGGAGTAAAACGACGGCCAGT	Reverse
M13F_6	GACGGAAGTAAAACGACGGCCAGT	Reverse
M13F_7	TTGCAGAGTAAAACGACGGCCAGT	Reverse
M13F_8	GAAGTCGGTAAAACGACGGCCAGT	Reverse
M13F_9	GCTGCTTGTAAAACGACGGCCAGT	Reverse
M13F_10	GTGACAAGTAAAACGACGGCCAGT	Reverse
M13F_11	GAACCAAGTAAAACGACGGCCAGT	Reverse
M13F_12	GTAATGCGTAAAACGACGGCCAGT	Reverse
